# Conflict of interest in academic oncology: moving beyond the blame game and forging a path forward

**DOI:** 10.1038/bcj.2016.101

**Published:** 2016-11-04

**Authors:** V Prasad, S V Rajkumar

**Affiliations:** 1 Division of Hematology Oncology, Department of Public Health and Preventive Medicine, Knight Cancer Institute, Oregon Health & Science University, Portland, OR, USA; 2 Division of Hematology, Department of Medicine, Mayo Clinic, Rochester, MN, USA

The management of financial conflict of interest (COI) in academic medicine has again been thrust in the spotlight,^1^ with some writers contending that current COI policies—largely a mandate to disclose conflicts, and, on rare occasion, recusal from academic opportunities or divestiture of the conflict—may perversely stifle biomedical innovation.^2, 3, 4, 5^ Others have argued in opposition, noting that loss of innovation is speculative, that historical breaches of trust led to current policies, and that the bias of financial COI may be subtle.^6^ Although there are clearly many types of conflict—consistency with prior views, desire for success, preference for friends or family, organizational and professional allegiances—financial conflicts have been highlighted over the last three decades in part because they are objectively measurable, have documented associations with favorable and positive results and in part because they are unidirectional—while other conflicts sometimes point for or against some treatment or diagnostic test—financial conflicts are nearly always aligned with more care.^7^

The role financial COI plays in oncology, especially in the United States, has not been discussed. Interactions between academic investigators and the biopharmaceutical industry are particularly important in oncology for three reasons: (1) many treatment decisions in oncology are based on ‘expert' interpretation of suboptimal data, (2) a considerable amount of therapy involves off-label use of highly expensive treatments and (3) the majority of new drug approvals now occur in oncology, and interactions are common.^8^ For these reasons, we believe the nature and appropriate management of oncology COI deserves special consideration. At the same time, we acknowledge the considerations here apply to other fields or subfields with similar advances in therapeutics and large industry presence, and our solutions may extend outside of oncology.

## The true north of academic medicine

Undoubtedly, the for-profit motivation of the industry is a great driver of innovation. When the profit motive is added to human endeavors it nearly always spurs activity, and harnesses energy and industry that may be lost in non-profit or governmental systems. If medical progress is a car, use of the profit motive is a foot on the gas pedal.

At the same time, the profit motive does not always point in the exact same direction as the ultimate goal of health care and academic medicine. The ultimate interest of academic medicine is to improve human health as much as possible, through treatments with as little toxicity as possible, and, all else being equal, for as low a price to patients and society as possible. The goal of industry may often be aligned with this, but in some settings may be slightly tangential. If the true goal of academic medicine is labeled ‘true north', the ultimate goal of industry—promotion of their products—may be called ‘magnetic north', in analogy to the globe. The analogy may be considered particularly apt, as one considers the draw or pull of the magnetic north.

Consider, for instance, a cancer drug with marginal benefits and real toxicity. Such a medication may rightly be debated by academics, but the industry's goal is overwhelmingly to lobby for approval and use. Consider the simple fact that while the US Food and Drug Administration's Oncology Drug Advisory Committee (ODAC) votes against approval 48% of the time,^9^ no sponsoring company has ever argued against approval at an ODAC meeting. If the arrows were perfectly aligned, we might expect less discordance. Thus, the benefit of enhanced acceleration from for-profit involvement in medicine may be offset very slightly by a misalignment in the steering column. Of course, such a trade-off is both reasonable and acceptable, and often manageable, but the key is having a system in place to redirect the vehicle.

## Financial conflict in academic oncology

Many experts in academic oncology work with pharmaceutical companies, and have substantial financial conflicts including, but not limited to, service on advisory boards, ownership of equity (or stock options), patent royalties, consulting fees and honoraria for speaking (https://projects.propublica.org/docdollars/). An examination of the reported conflicts of interest by experts presenting lectures at major medical meetings reveals few (~30%) who declare ‘no competing interests'.^10^ The problem is that such conflicts can influence treatment practices more extensively and can inflict cost and potentially toxicity to a greater deal in oncology than in other fields.

First, for many major treatment decisions in oncology, there is no standard-of-care established by phase III randomized trials. For example, although the initial therapy of diffuse large cell lymphoma may be a settled issue (for the moment), many subsequent therapies are based on lower levels of evidence that require ‘expert' interpretation. In fact, in many cancers, there are no phase III trials that have clearly established a given regimen as standard-of-care for initial therapy (over available alternatives), let alone for subsequent treatment decisions.

Second, although some oncologic drugs are truly transformative, many others are merely marginal^11^ often with real toxicity. And because so much of cancer decision-making, guidelines writing and drug approval involves the careful balancing of risks and benefits—in other words, is gray—the role of direct financial conflicts, even if only subtle and only present in every third physician, it can lead to decisions that may not be perfectly oriented with the best patient care. In many tumor types, there is at least one drug that may only improve a surrogate endpoint marginally, with significant toxicity or intolerability, in a trial with heavy and perhaps differential censoring^12^—and even these sobering results occurred in a carefully selected patient population,^13^ and are likely to be more unfavorable in the real world. In short, in each field of oncology, we have at least one truly difficult question of whether it is worth it to prescribe some particular drug as a general policy—and these are the cases in which one worries about the role of financial conflict.

Third, the influence of leaders at the very top of the field can be substantial and disproportionate in oncology, especially as we parse out cancers into smaller and smaller categories—collections of rare diseases. A recommendation from one or two of the leading experts can dramatically change what is perceived as ‘standard-of-care'. Finally, the potential economic cost of a recommendation in oncology can be staggering.^14, 15^ For example, almost all new drugs in oncology are priced over $100 000 per patient per year;^15^ any recommendation concerning how and when to use one of these drugs (often, off-label) will have a huge impact on health care cost to the individual patient and society.

Like others, we do not assume that financial conflicts inherently play a role in such decisions, but merely acknowledge that, ‘it is often difficult if not impossible to distinguish cases in which financial gain does have improper influence from those in which it does not'.^16^ These conflicts in oncology are particularly problematic in the area of continuing medical education (CME), the development and dissemination of practice guidelines, and with regards to the role of professional/patient organizations.

## Continuing medical education

Although we admit to greatly enjoying professional conferences, concerns have been raised about these meetings.^17^ Conferences permit the presentation of abstracts, some of lesser quality, because the primary goal in many instances is mainly to generate hypothesis, and not meant to immediately influence practice. Medical conferences shape the early debate and discussion of novel therapeutics or analytics, but by nature disseminate preliminary data that is not fully peer-reviewed. Further, approximately a third of randomized trials, and approximately half of other study designs, are never subsequently presented in full manuscript form.^18^ Despite these caveats, information presented at major conferences frequently forms the basis for clinical decisions. Some of this, such as presentation of life-saving data from randomized trials is indeed justified, but it is likely that some information derived from conferences is used prematurely in the clinic.

Industrial presence is ubiquitous at oncology conferences, with large exhibits showcasing the drug ‘pipeline' and representatives eager to shape one's impression of drugs in development or those newly approved. News coverage in the wake of the national meetings hails many approved and unapproved drugs as ‘game changers' or ‘miracles'.^19^ In short, the meeting itself permits corporate sponsors opportunity for repeated academic interaction in a setting in which the audience is already primed to savor preliminary data presented at oral and poster sessions.

Industry funding of CME, satellite symposia or dinner lectures are ways in which the education of physicians may be subtly influenced. Although the use of company-sponsored CME appears to have peaked in 2007, it remains common.^20^

## Practice guidelines

The majority of recommendations in oncology guidelines—such as the National Comprehensive Cancer Network—are made on the basis of non-RCT evidence.^21^ Moreover, the choice of regimens listed for the treatment of a particular malignancy is often a ‘laundry-list' of all possible regimens that could be used in that setting. This in part occurs because reimbursement is tied to inclusion in compendia. And when there are 10 ‘reasonable' options, it results in a ‘free-for-all' when it comes to what treatment a given patient will actually receive in practice. Every company with a product in a given disease wants to be on that list, and we presume that through CME or otherwise once a regimen makes the list, it will somehow find its way into clinical practice. Data suggest that >80% of National Comprehensive Cancer Network authors have personal financial payments from companies.^22^ The problem unique to oncology is that most cancer therapy can be considered experimental, and guidelines rather than evidence are the driver of most clinical decisions. Together, these facts prompt concern. As guidelines involve decision-making in uncertain circumstances, and because conflict is common among writers, we see instances where guidelines err on the side of promoting drugs for indications of uncertain risk and benefit. It must be acknowledged that it is unclear if the guidelines would be different if these conflicts were not present, however, in many cases we believe that they would be.

## The deep conflicts

Besides direct financial conflicts of interest, there are other deeper conflicts that are built into the oncology system perhaps in a more systemic manner than in other fields of medicine. The reputation, and in some countries, the livelihood, of academic clinical oncologists is partly dependent on conducting, participating or leading clinical trials. These trials often require industry partnership. For instance, between 2005 and 2009, 78% of randomized trials in oncology received industry support.^23^ Even collaboration between the federally funded cooperative groups and the industry is more common today than even 15 years ago.^24^ And, while such collaboration is essential, it does present two conflicts. The first occurs at the outset of the trial: the precise wording of the clinical protocol is often decided by or has significant input from the industry.^25^ Thus, academics, on occasion, have to accept some design features that may point slightly away from the true north to be a lead investigator on the trial—these features are described at length elsewhere.^26^

The second occurs in certain cases at the end of the trial: industry-sponsored trials may employ medical writers to provide the lead investigator with the first draft of the manuscript, and the key conclusions. The role of writing assistance for medical manuscripts may improve the readability, and spares significant time for busy academics,^27^ yet when provided directly by industry, it can highlight the benefits and harms of drugs in ways that may be distinct from how academics would choose do so. Writing assistance remains common with 21% of authors of articles in the 6 highest impact factor medical journals reporting honorary or ghost authorship.^28^ Industry-sponsored trials fair worse than this estimate with 75% containing some ghost authorship.^29^ The conflict is that a busy academic can either choose to have a prominent publication with little effort, or struggle on his or her own. If medical writing assistance is accepted, it is more difficult to challenge the sponsor on a debatable conclusion, spin or overemphasis of a secondary endpoint.

As with direct financial conflicts, we do not believe that these conflicts inherently lead to false information or misrepresentations of the truth. It is just that it is impossible for the reader of the literature to determine whether or not such tensions exist.

## Solutions

The problems we have outlined convey the sobering fact that at many junctures in oncology, conflicts of interest may exert a soft but steady pull in a direction slightly tangential from best patient care. The problem is not unique to oncology, but as discussed earlier may be more extensive and have greater impact on practice compared with most other fields of medicine. There are no easy solutions, and some of our proposed solutions may not be palatable to all. But we owe it to our patients to begin a conversation. We propose 4 first-steps that are easy to enforce and will go a long way in removing the cloud of COI that hangs over our field.

First, we propose that authors of reviews, editorials and practice guidelines—the areas of oncology where professional judgment plays a guiding role—should not have any financial COI that exceeds the de-minimis threshold ($10 000 per calendar year per company) proposed in the *New England Journal of Medicine.*^30^ Speakers at the educational sessions at major society meetings (American Society of Hematology, American Society of Clinical Oncology, European Hematology Association) should also fall under these rules. These individuals exert an extraordinary influence over how cancer is treated and they need to be free of substantial financial conflicts. Payments for the actual conduct of a clinical trial and payments from multi-company-sponsored CME meetings would be excluded. But payments from single-company-sponsored CME meetings, consulting fees, patent royalties, direct remuneration from employment and stock options would be included. We urge major Journals (such as the *New England Journal of Medicine*, *The Lancet*, *The Lancet Oncology*, *The Journal of the American Medical Association* (JAMA), *JAMA Oncology*, *the Journal of Clinical Oncology*, *Blood* and *Leukemia*) and major professional organizations (such as the American Society of Hematology, American Society of Clinical Oncology and National Comprehensive Cancer Network) to adopt these rules.

Second, we recommend that we stop the practice of using professional medical writers who are funded by industry for any manuscript. Authors are probably not aware that many companies report as payment (transfer of value) against their name to the Sunshine Act website when a medical writer is involved in the preparation of a manuscript. If an author requires a professional writer, such a person or group should be hired using funds from the academic institution, or using personal funds. This is something that can be easily enforced by Journals.

Third, we urge organizations to make fully transparent the extent of industry support at our meetings and our work. We believe transparency may prompt some reconsideration of the extent of support. At a recent medical meeting, in the exhibitor hall amidst lavish pharmaceutical displays, the exhibits one would actually want doctors to visit (medical books) were languishing in the most remote corner. Transparency is key, and how much payments each organization received from each specific pharmaceutical company in a calendar year should be made publicly available to the membership of the organization, and readily available at medical meetings. As with individual physicians, the problem is not that there are no safeguards and firewalls to ‘prevent' the choice of topics at the meeting to be influenced by conflicts. It is the fact that no one can really tell whether a conflict exists or not, and disclosure is best.

## Conclusion

COI refers to any motivation that changes the compass of academic medicine, and leads us to pursue or embrace treatments or diagnostics that are not the absolute best (given the practical realities of cancer care) for our patients. Some drugs may have questionable risk-benefit profiles, or their use in preclinical or early disease states may be questioned—and at the same time using these drugs may be desired by the industry—setting the tension for COI.

The present system in oncology has many structural elements that amplify the problem more so than other fields of medicine. Conflict is always subtle, and the majority of doctors may not be influenced, while others may be, but may not believe they are being influenced. The problem is that the user can never find out who is and who is not conflicted. Merely reporting conflicts would, at present, be largely adequate for the presentation of original research where the reader/user can evaluate the raw data themselves. But when it comes to expert opinion driven practice (reviews, editorials, guidelines and so on), declaring conflicts is not enough. There simply cannot be room for significant conflict.

We understand that the solutions we propose can have unintended consequences. Some are already enforced by the *New England Journal of Medicine*, and some other journals have stricter rules.^31^ We do not think that our proposals will deprive us of the most-qualified experts. Experts who have significant financial conflicts can always shed their conflicts if they chose to; or provide input to those authors/experts who are not conflicted.

There is no role in this debate for blame. Nevertheless, we must think deeply about how current oncologic care deviates from the true mission of academic medicine, and we must slowly but surely eliminate structural factors that prevent us from moving unwaveringly in the right direction.

## References

[bib1] Cappola AR, FitzGerald GA. Confluence, not conflict of interest: name change necessary. JAMA 2015; 314: 1791–1792. 26402846 10.1001/jama.2015.12020

[bib2] Rosenbaum L. Beyond moral outrage — weighing the trade-offs of COI regulation. N Engl J Med 2015; 372: 2064–2068. 25992752 10.1056/NEJMms1502498

[bib3] Rosenbaum L. Reconnecting the dots — reinterpreting industry–physician relations. N Engl J Med 2015; 372: 1860–1864. 25946288 10.1056/NEJMms1502493

[bib4] Rosenbaum L. Understanding bias — the case for careful study. N Engl J Med 2015; 372: 1959–1963. 25970055 10.1056/NEJMms1502497

[bib5] Drazen JM. Revisiting the commercial-academic interface. N Engl J Med 2015; 372: 1853–1854. 25946285 10.1056/NEJMe1503623

[bib6] Steinbrook R, Kassirer JP, Angell M. Justifying conflicts of interest in medical journals: a very bad idea. BMJ 2015; 350: h2942. 26036926 10.1136/bmj.h2942

[bib7] Lenzer J, Brownlee S. Diverting attention from financial conflicts of interest. BMJ 2015; 350: h3505. 26129927 10.1136/bmj.h3505

[bib8] Mullard A. 2013 FDA drug approvals. Nat Rev Drug Discov 2014; 13: 85–89. 24481294 10.1038/nrd4239

[bib9] Chan JK, Kiet TK, Monk BJ, Young-Lin N, Blansit K, Kapp DS et al. Applications for oncologic drugs: a descriptive analysis of the oncologic drugs advisory committee reviews. The Oncologist 2014; 19: 299–304. 24599479 10.1634/theoncologist.2013-0276 PMC3958455

[bib10] Boothby A, Wang R, Cetnar J, Prasad V. Effect of the American Society of Clinical Oncology's Conflict of Interest Policy on Information Overload. JAMA Oncol, e-pub ahead of print 25 August 2016 doi:10.1001/jamaoncol.2016.2706. 10.1001/jamaoncol.2016.2706 27560393

[bib11] Fojo T, Mailankody S, Lo A. Unintended consequences of expensive cancer therapeutics—the pursuit of marginal indications and a me-too mentality that stifles innovation and creativity: The john conley lecture. JAMA Otolaryngol Head Neck Surg 2014; 140: 1225–1236. 25068501 10.1001/jamaoto.2014.1570

[bib12] Prasad V, Bilal U. The role of censoring on progression free survival: oncologist discretion advised. Eur J Cancer 2015; 51: 2269–2271. 26259493 10.1016/j.ejca.2015.07.005

[bib13] Talarico L, Chen G, Pazdur R. Enrollment of elderly patients in clinical trials for cancer drug registration: a 7-Year experience by the US Food and Drug Administration. J Clin Oncol 2004; 22: 4626–4631. 15542812 10.1200/JCO.2004.02.175

[bib14] Siddiqui M, Rajkumar SV. The high cost of cancer drugs and what we can do about it. Mayo Clin Proc 2012; 87: 935–943. 23036669 10.1016/j.mayocp.2012.07.007 PMC3538397

[bib15] Mailankody S, Prasad V. Five years of cancer drug approvals: Innovation, efficacy, and costs. JAMA Oncol 2015; 1: 539–540. 26181265 10.1001/jamaoncol.2015.0373

[bib16] Thompson DF. Understanding financial conflicts of interest. N Engl J Med 1993; 329: 573–576. 8336759 10.1056/NEJM199308193290812

[bib17] Ioannidis JA. Are medical conferences useful? and for whom? JAMA 2012; 307: 1257–1258. 22453564 10.1001/jama.2012.360

[bib18] Scherer RW, Langenberg P, von Elm E. Full publication of results initially presented in abstracts. Cochrane Database Syst Rev 2007; 2: MR000005. 10.1002/14651858.MR000005.pub3 17443628

[bib19] Abola MV, Prasad V. The use of superlatives in cancer research. JAMA Oncol 2016; 2: 139–141. 26512913 10.1001/jamaoncol.2015.3931

[bib20] Steinman MA, Landefeld CS, Baron RB. Industry support of CME — are we at the tipping point? N Engl J Med 2012; 366: 1069–1071. 22435367 10.1056/NEJMp1114776

[bib21] Poonacha TK, Go RS. Level of scientific evidence underlying recommendations arising from the national comprehensive cancer network clinical practice guidelines. J Clin Oncol 2011; 29: 186–191. 21149653 10.1200/JCO.2010.31.6414

[bib22] Mitchell AP, Basch EM, Dusetzina SB. Financial Relationships With Industry Among National Comprehensive Cancer Network Guideline Authors. JAMA Oncol, e-pub ahead of print 25 August 2016; doi:doi:10.1001/jamaoncol.2016.2710. 10.1001/jamaoncol.2016.2710 27561170

[bib23] Kay A, Higgins J, Day AG, Meyer RM, Booth CM. Randomized controlled trials in the era of molecular oncology: methodology, biomarkers, and end points. Ann Oncol 2012; 23: 1646–1651. 22048151 10.1093/annonc/mdr492

[bib24] Bressler LR, Schilsky RL. Collaboration Between cooperative groups and industry. J Oncol Pract 2008; 4: 140–141. 20856620 10.1200/JOP.0834601 PMC2793998

[bib25] Piccart M, Goldhirsch A, Wood W, Pritchard K, Baselga J, Reaby L et al. Keeping faith with trial volunteers. Nature 2007; 446: 137–138. 17344833 10.1038/446137a

[bib26] Prasad V, Berger VW. Hard-wired bias. Mayo Clin Proc 2015; 90: 1171–1175. 26277702 10.1016/j.mayocp.2015.05.006 PMC4567484

[bib27] Marchington JM, Burd GP. Author attitudes to professional medical writing support. Curr Med Res Opin 2014; 30: 2103–2108. 25007242 10.1185/03007995.2014.939618

[bib28] Wislar JS, Flanagin A, Fontanarosa PB, DeAngelis CD. Honorary and ghost authorship in high impact biomedical journals: a cross sectional survey. BMJ 2011; 343: d6128. 22028479 10.1136/bmj.d6128 PMC3202014

[bib29] Gøtzsche PC, Hróbjartsson A, Johansen HK, Haahr MT, Altman DG, Chan A-W. Ghost authorship in industry-initiated randomised trials. PLoS Med 2007; 4: e19. 17227134 10.1371/journal.pmed.0040019 PMC1769411

[bib30] Drazen JM, Curfman GD. Financial associations of authors. N Engl J Med 2002; 346: 1901–1902. 12063375 10.1056/NEJMe020074

[bib31] Chew M, Brizzell C, Abbasi K, Godlee F. Medical journals and industry ties. BMJ 2014; 349: g7197. 25432164 10.1136/bmj.g7197

